# The persistence and mortality of Lassa fever in Nigeria reflect systemic clinical and diagnostic challenges rather than viral reemergence alone

**DOI:** 10.3389/fepid.2026.1837721

**Published:** 2026-06-17

**Authors:** Opeyemi Hammed, Miracle Adesina, Busayo Buoye, Damilare Taiwo, Isaac Olufadewa, Dorcas Alabi

**Affiliations:** 1 Center for One Health and Zoonotics Diseases (COHAZD), Slum and Rural Health Initiative (SRHIN), Ibadan, Oyo State, Nigeria; 2 Department of Microbial Genetics and Biotechnology, Covenant University, Ota, Ogun State, Nigeria; 3 Acute Assessment Unit, Royal Sussex County Hospital, Brighton, United Kingdom

**Keywords:** diagnostic delay, health system capacity, Lassa fever, respiratory and systemic complication, viral mutation

## 1 Introduction

Each seasonal surge of Lassa fever deaths reignites a familiar question in Nigeria: has the virus become more dangerous?. The evidence suggests otherwise. Despite decades of clinical familiarity with the Lassa virus, mortality persists, driven less by viral evolution than by delayed recognition, limited diagnostic access, and constrained supportive care (1, 2).

Patients frequently present after days of treatment for presumed malaria or typhoid fever (3), reaching referral centres only when complications acute kidney injury (4), respiratory distress (5), or hemorrhagic manifestations have advanced beyond reversible stages (6). This recurring pattern accentuates a critical reality: in endemic settings, survival is determined not solely by pathogen's biology but by the timing, accuracy, and capacity of clinical response. Lassa fever remains endemic in Nigeria and continues to pose a significant clinical and public health burden (7). Seasonal peaks, typically during the dry season, are driven by increased human contact with rodent reservoirs and heightened household exposure risks (8). Although surveillance and treatment centres have expanded over the past decade, case fatality rates remain substantial among hospitalized patients. Public discourse often frames annual surges as “reemergence,” implying viral change or increased virulence. However, epidemiological patterns instead reflect persistent transmission dynamics within vulnerable communities and health systems that remain unevenly equipped to ensure early detection and timely care (9). Understanding why mortality remains high despite long-standing awareness and established treatment protocols is critical. Framing Lassa fever as a reemerging viral threat risks diverting attention from modifiable clinical and system-level determinants that directly influence survival.

## 2 Clinical reality of Lassa fever and diagnostic challenges and clinical mimicry

Clinically, Lassa fever presents along a spectrum ranging from mild febrile illness to severe multisystem disease (10). Early symptoms, fever (11), malaise (12), sore throat (13), headache (14), vomiting (12), and diarrhea (15) are nonspecific and overlap with common endemic infections. In severe cases, patients may develop mucosal bleeding, facial edema, encephalopathy, acute kidney injury, respiratory compromise, and shock (16, 17). Sensorineural hearing loss, a hallmark sequela, affects a substantial proportion of survivors and contributes to long-term disability (14). In practice, many patients present at referral centres with advanced disease. Severe complications frequently reflect delayed diagnosis and late initiation of supportive care rather than intrinsic viral virulence (18). This clinical trajectory highlights the central importance of early recognition and timely intervention.

One of the most significant barriers to survival is the difficulty of distinguishing Lassa fever from other febrile illnesses in early stages. Malaria, typhoid fever, and bacterial sepsis remain more common and are often treated empirically before Lassa fever is suspected (19). This overlap delays clinical suspicion and postpones infection prevention measures and antiviral therapy. Diagnostic capacity further complicates early detection. Confirmatory testing relies largely on reverse transcription polymerase chain reaction (RT-PCR), a highly sensitive and specific method typically conducted at specialized laboratories (20). However, in many endemic areas, access to such facilities is limited and samples must be transported over long distances, leading to delays in confirmation. The absence of widely available, reliable point-of-care diagnostics limits clinicians' ability to triage suspected cases promptly. As a result, treatment decisions are often made in uncertainty, and patients may not receive appropriate isolation or targeted care until disease progression is advanced (21).

## 3 Timing of presentation and disease severity and health system and clinical infrastructure gaps

Late presentation to healthcare facilities is a consistent feature of severe Lassa fever cases. Patients frequently seek care initially at local drug vendors or primary facilities, where treatment for malaria or bacterial infections is initiated (22). Geographic barriers, financial constraints, and limited awareness further delay referral to specialized centres. By the time patients arrive at treatment centres, viral load may be high and organ dysfunction established (23). Acute kidney injury, respiratory failure, and circulatory collapse significantly increase mortality risk. Evidence consistently demonstrates that early antiviral therapy and supportive care improve outcomes, underscoring the clinical consequences of delayed presentation (24). Health system limitations amplify mortality risk. Many facilities lack dedicated isolation units, adequate personal protective equipment, and sufficient infection prevention and control infrastructure (25). Delayed recognition of suspected cases increases the risk of nosocomial transmission and healthcare worker exposure. Supportive care capacity remains uneven. Management of severe Lassa fever requires careful fluid balance, oxygen therapy, renal support, and continuous monitoring (26). However, shortages of dialysis services, oxygen delivery systems, and intensive care resources reduce survival chances for critically ill patients. Workforce constraints and limited training in early triage and viral hemorrhagic fever preparedness further hinder effective response (27). Certain populations face disproportionately high risks. Pregnant women experience markedly elevated mortality and fetal loss rates. Children may present atypically, complicating early recognition. Individuals with comorbidities, malnutrition, or compromised immune status are more vulnerable to severe disease (28). These disparities reflect broader inequities in access to timely and quality healthcare.

## 4 Reframing the narrative: beyond viral mutation

The Lassa virus exhibits genetic diversity, and multiple lineages circulate within Nigeria, while viral evolution may influence transmission dynamics and disease severity, current evidence does not support mutation as the primary explanation for persistent mortality (29). Rather, endemicity, environmental exposure, delayed diagnosis, and constrained clinical capacity provide a more compelling explanation. Reframing the narrative away from viral reemergence toward systemic clinical determinants redirects attention toward actionable solutions capable of reducing preventable deaths. Reducing mortality requires strengthening frontline clinical response (30). Clinician awareness and training should emphasize early suspicion of Lassa fever in patients with persistent fever unresponsive to antimalarial therapy (31). Standardized triage algorithms for febrile illness can support early identification and prompt isolation. Decentralizing rapid diagnostic testing and improving specimen transport systems would shorten time to confirmation and treatment initiation. Strengthening referral networks and emergency transport systems can reduce delays in accessing specialized care (32). Equally critical is investment in supportive care capacity, including oxygen therapy, renal support services, and patient monitoring. Enhancing infection prevention infrastructure protects healthcare workers and reduces nosocomial transmission (33). Addressing persistent mortality requires targeted research aligned with clinical realities. Priority areas include development of clinical prediction tools to support early diagnosis, validation of point-of-care diagnostic technologies, and identification of biomarkers predictive of disease severity. Operational research on supportive care protocols in resource-limited settings is essential to optimize survival (34). Long-term survivorship research is also needed, particularly regarding hearing loss, neurocognitive outcomes, and chronic renal impairment (35). Implementation science approaches can evaluate strategies to integrate rapid diagnostics, strengthen triage systems, and improve referral pathways in endemic regions.

Despite arguments against viral mutation as the primary driver of mortality, several factors contribute to the *perceived reemergence* of Lassa fever in Nigeria. Ecological and environmental changes play a significant role, particularly increased human interaction with the rodent reservoir (*Mastomys natalensis*) due to deforestation, agricultural expansion, and poor housing conditions (36). Seasonal patterns, especially during the dry season, further increase exposure risk as rodents migrate into human dwellings in search of food and shelter. Rapid urbanization and population growth in endemic regions have also intensified human–rodent contact (37). Additionally, improvements in disease surveillance, reporting systems, and diagnostic capacity have led to increased detection and documentation of cases, which may be misconstrued as reemergence rather than improved case ascertainment. Population mobility, including rural–urban migration and cross-border movement, further facilitates the spread of infection (38). Persistent gaps in infection prevention and control practices, as well as limited community awareness, also sustain transmission cycles. These factors reflect structural and environmental dynamics rather than fundamental changes in viral pathogenicity. Recognizing these drivers supports a more accurate interpretation of Lassa fever epidemiology and reinforces the need for integrated public health and clinical responses.

Beyond clinical and health system challenges, community-level factors play a critical role in sustaining Lassa fever transmission. Poor housing conditions, inadequate food storage practices, and limited environmental sanitation increase human exposure to the primary rodent reservoir, *Mastomys natalensis* (39). Additionally, low awareness of Lassa fever symptoms and transmission pathways contributes to delayed care-seeking and continued community spread. These factors highlight the importance of community engagement as a central component of Lassa fever control. Public health education, culturally appropriate risk communication, and community-based interventions aimed at improving housing, sanitation, and rodent control are essential to reducing exposure and interrupting transmission cycles (40). Integrating these strategies with strengthened clinical response will provide a more comprehensive approach to reducing both incidence and mortality.

## 5 Conclusion

Lassa fever continues to claim lives in Nigeria not because the pathogen is new, but because timely diagnosis and effective clinical care remain unevenly accessible. Persistent mortality reflects systemic clinical and diagnostic gaps that delay life-saving interventions. Reframing Lassa fever as a challenge of health system readiness and clinical responsiveness rather than viral reemergence alone highlights opportunities to reduce preventable deaths. Strengthening early recognition, expanding diagnostic access, and improving supportive care capacity will not only improve survival from Lassa fever but also enhance preparedness for other viral hemorrhagic fevers in resource-limited settings.
